# Supplementary material for: MetaRibo-Seq measures translation in microbiomes
Source: Nat Commun. 2020 Jun 29;11:3268. doi: 10.1038/s41467-020-17081-z (PMC7324362; doi:10.1038/s41467-020-17081-z)
Supplement: Supplementary file 10 — Supplementary Data 7 [file 41467_2020_17081_MOESM10_ESM.zip › File2/Confidence_High_Taxonomy/386917_out.krona.html]

Javascript must be enabled to view this page.

members
magnitude
magnitudeUnassigned
count
unassigned
taxon
rank

386917\_out

55

2
55
superkingdom

55
1239
phylum

class
55
186801

186802
55
4
order

SRS022713\_contig\_number\_7510SRS063040\_contig\_number\_contig-100\_845.251627SRS143780\_contig\_number\_29392SRS144135\_contig\_number\_8661

family

SRS012273\_contig\_number\_40814SRS014979\_contig\_number\_22217SRS019397\_contig\_number\_8168SRS042284\_contig\_number\_contig-100\_853.52314SRS057717\_contig\_number\_6669SRS146813\_contig\_number\_15535SRS147557\_contig\_number\_4366SRS147977\_contig\_number\_132SRS148721\_contig\_number\_19164
186803
51
9

genus
19
841

1638781
18
species

SRS013940\_contig\_number\_19255SRS013951\_contig\_number\_35641SRS014235\_contig\_number\_38031SRS015217\_contig\_number\_30815SRS015794\_contig\_number\_7371SRS017521\_contig\_number\_28950SRS042628\_contig\_number\_28847SRS043411\_contig\_number\_4455SRS103987\_contig\_number\_34815SRS1041091\_contig\_number\_5801SRS104485\_contig\_number\_4429SRS1055067\_contig\_number\_296SRS142712\_contig\_number\_5119SRS143342\_contig\_number\_16908SRS144603\_contig\_number\_2399SRS148091\_contig\_number\_12271SRS148159\_contig\_number\_40162SRS149244\_contig\_number\_4436

1
360807
species

SRS148253\_contig\_number\_12893

572511
12
genus

33039
12

SRS013965\_contig\_number\_40731SRS014613\_contig\_number\_14333SRS014736\_contig\_number\_733SRS014948\_contig\_number\_17788SRS015065\_contig\_number\_12395SRS016267\_contig\_number\_11859SRS016495\_contig\_number\_contig-100\_1372.114582SRS019601\_contig\_number\_28073SRS057478\_contig\_number\_7301SRS064645\_contig\_number\_contig-100\_601.601SRS097889\_contig\_number\_36766SRS098655\_contig\_number\_13252
species

1898203
3
species

SRS014287\_contig\_number\_778SRS043768\_contig\_number\_5338SRS146812\_contig\_number\_60659

658081
7

SRS014459\_contig\_number\_5836SRS014923\_contig\_number\_31915SRS063518\_contig\_number\_37580SRS077730\_contig\_number\_11504SRS098717\_contig\_number\_22351SRS142599\_contig\_number\_31019SRS142890\_contig\_number\_39906
species

189330
1
genus

species

SRS143991\_contig\_number\_27276
88431
1
